# SARS-CoV-2 Gamma Variant In Vivo Model Demonstrates Severe Pathogenicity and Unique Transcriptomic Signatures Compared to Alpha, Delta, and Omicron Variants

**DOI:** 10.3390/microorganisms14102400

**Published:** 2026-10-20

**Authors:** Fernanda Martins Marim, Paula Luize Camargos Fonseca, Maria Inês Mantuani Pascoaloti, Aryel Beatriz Paz e Silva, Fernanda Gil de Souza, Érika Donizetti Candido, Vivian Vasconcelos Costa Litwinski, Danielle Bruna Leal Oliveira, Edison Luiz Durigon, Rennan Garcias Moreira, Renan Pedra de Souza, Vanessa Fátima Bernardes, Renato Santana Aguiar

**Affiliations:** 1 Department of Genetics, Ecology and Evolution, Institute of 9Biological Sciences, Universidade Federal de Minas Gerais, Belo Horizonte 31270-901, MG, Brazil; fernanda_marim@yahoo.com.br (F.M.M.); camargos.paulaluize@gmail.com (P.L.C.F.); arypaz06@gmail.com (A.B.P.e.S.); nandags10@hotmail.com (F.G.d.S.);; 2 Department of Pathology, Institute of Biological Sciences, Universidade Federal de Minas Gerais, Belo Horizonte 31270-901, MG, Brazil; mariainespsc@outlook.com; 3 Department of Microbiology, Institute of Biomedical Sciences, Universidade de São Paulo, São Paulo 05508-000, SP, Brazil; erikacandido@usp.br (É.D.C.); eldurigo@usp.br (E.L.D.); 4 Department of Morphology, Institute of Biological Sciences, Universidade Federal de Minas Gerais, Belo Horizonte 31270-901, MG, Brazil; vivianvcosta@gmail.com; 5 Laboratório de Virologia Clínica e Molecular, Instituto de Ciências Biomédicas, Universidade de São Paulo, São Paulo 05508-000, SP, Brazil; danibruna@usp.br; 6 Hospital Israelita Albert Einstein, São Paulo 05652-000, SP, Brazil; 7 Genomic Laboratory, Universidade Federal de Minas Gerais, Belo Horizonte 31270-901, MG, Brazil; rennangm@gmail.com; 8 Instituto René Rachou, Fundação Oswaldo Cruz, Belo Horizonte 30190-002, MG, Brazil; 9 Instituto D`OR de Pesquisa e Ensino, Rio de Janeiro 22281-100, RJ, Brazil

**Keywords:** SARS-CoV-2, variants of concern, K18-hACE2 mice, pulmonary pathogenesis, histopathology, immunohistochemistry, transcriptomic profiling

## Abstract

Since 2019, SARS-CoV-2 variants of concern (VOCs), Alpha, Gamma, Delta, and Omicron, have exhibited distinct transmissibility and severity profiles. In Brazil, Gamma caused high mortality pre-vaccination, yet its underlying pathological mechanisms remain poorly understood. We evaluated VOC pathogenesis in K18-hACE2 transgenic mice (*n* = 8/group) by integrating clinical monitoring, lung histopathology, immunohistochemistry (F4/80, Ly-6G, CD4, CD8, Spike), and transcriptomics. Alpha, Gamma, and Delta induced severe weight loss, high clinical scores, and 100% mortality within seven days, whereas Omicron caused milder outcomes and survival despite similar pulmonary viral loads (10^7^–10^8^ copies/µL). Histopathology showed intense inflammation and hyperemia across all VOCs, with hierarchical clustering revealing a unique profile for Gamma. Transcriptomics identified 117 core upregulated genes, but Alpha and Gamma modulated a broader gene set. Gamma upregulated components of the alternative complement pathway (*Cfb*), heightened inflammatory mediators, and downregulated tissue adhesion genes. These results demonstrate that the Gamma variant phenotype is driven by extensive transcriptomic remodeling, whereas Omicron elicits an attenuated response, providing key insights into variant-specific mechanisms.

## 1. Introduction

Since its emergence in late 2019, Severe Acute Respiratory Syndrome Coronavirus 2 (SARS-CoV-2) has caused an unprecedented global public health crisis. Continuous viral evolution has led to the emergence of multiple Variants of Concern (VOCs), which have been associated with increased transmissibility, enhanced disease severity, and altered clinical outcomes. The major VOCs identified to date include Alpha, Gamma, Delta, and Omicron, which emerged at different time points during the pandemic [1,2].

The Alpha variant (B.1.1.7) was first identified in the United Kingdom in late 2020. It rapidly became the dominant lineage there and in other regions, due to its higher transmissibility compared with previously circulating strains. Early epidemiological analyses also reported increased risks of hospitalization and mortality associated with Alpha infections relative to earlier lineages [3,4,5]. Although Alpha was detected in Brazil during the same period, its prevalence remained below 10%, in contrast to its widespread circulation in several Northern Hemisphere countries [6]. The Gamma variant (P.1), initially detected in Brazil, rapidly spread across South America and was associated with increased transmission and severe clinical manifestations in Brazil [7,8,9], with the highest number of deaths mainly in the Amazon region [8]. The circulation of both Alpha and Gamma declined following the widespread implementation of vaccination campaigns [10]. In contrast, the Delta (B.1.617.2) variant caused large outbreaks in multiple regions worldwide even after the initiation of mass vaccination programs, suggesting partial evasion of vaccine-induced immunity [2,11,12,13]. Subsequently, the Omicron variant (B.1.1.529), first identified in South Africa, rapidly disseminated across continents and was associated with a sharp increase in case numbers, despite generally milder clinical outcomes at the population level [2,12,13,14,15,16].

Collectively, these VOCs exhibit in their transmissibility, capacity for immune evasion, and associated clinical phenotypes [2]. Understanding how distinct SARS-CoV-2 variants interact with the host and modulate disease severity at the molecular and tissue levels remains a central challenge in COVID-19 research. Clinical and epidemiological studies have suggested that these VOCs are associated with distinct disease phenotypes, ranging from severe respiratory failure to milder clinical presentations [15,16]. However, the mechanisms underlying variant-specific pathogenicity and host responses remain incompletely understood.

The Gamma variant is of a particular interest in the Brazilian context [17,18]. Genomic and epidemiological analyses indicated that the rapid expansion of this variant contributed to the increased transmission observed during this period, although the magnitude and severity of the epidemic wave were also influenced by population immunity, demographic factors and public health structure [8,18,19]. These observations highlight the difficulty of dissociating variant-intrinsic pathogenic properties from the effects of existing immunity and other population-level factors in human studies. Therefore, experimental animal models provide an important complementary approach for investigating the biological properties of SARS-CoV-2 variants.

Transgenic K18-hACE2 mice represent a well-established experimental model for studying SARS-CoV-2 infection, as they present human ACE2 expression, enabling productive viral infection and the development of pathological alterations similar to those observed in severe human disease [20]. The K18-hACE2 model has been used to investigate differences in pathogenicity among SARS-CoV-2 variants under controlled experimental conditions [20,21]. Studies comparing Alpha and other early SARS-CoV-2 variants demonstrated that Alpha can induce severe disease and more pronounced tissue pathology in K18-hACE2 mice, accompanied by distinct inflammatory and cytokine responses [22]. Similarly, experimental infection with the Gamma variant has been associated with substantial weight loss, more pronounced clinical manifestations, impaired respiratory function and marked pulmonary pathology, with the Gamma exhibiting a more severe phenotype than the wild-type and Delta in one comparative study [23,24]. Experimental studies in K18-hACE2 mice have also shown that Delta infection can induce substantial pulmonary inflammation and tissue pathology despite viral RNA load comparable to those observed following Alpha infection, suggesting that differences in host responses may contribute to variant-specific disease phenotypes [23]. In contrast, several studies have reported the attenuated pathogenicity of Omicron in K18-hACE2 mice, including reduced viral replication in the respiratory tract, mild pulmonary pathology, lower body-weight loss and improved survival compared with earlier VOCs [25,26].

Although several studies have characterized the pathogenicity of individual SARS-CoV-2 variants, comparative analyses integrating lung pathology, immune cell infiltration, and global host gene expression across multiple VOCs at the same level of infection remain scarce. Transcriptomic analyses can capture coordinated changes in host gene expression associated with innate antiviral response, inflammatory signaling, immune cell recruitment, vascular injury, tissue damage and cellular repair, providing a molecular context for pathological phenotypes that may not be apparent from viral load alone. In particular, the relationship between variant-specific transcriptional programs and the extent of pulmonary damage has not been fully elucidated [2,16,27].

The present study therefore aimed to provide a controlled experimental comparison of Gamma with Alpha, Delta, and Omicron BA.1, focusing on variant-associated differences in clinical disease, pulmonary pathology, viral RNA load, and host transcriptional responses. By minimizing population-level confounding factors inherent to human epidemiological studies, this approach may provide insight into biological features associated with variant-specific disease phenotypes, without implying a direct explanation for differences in human mortality. Our results revealed substantial differences in disease manifestation and host transcriptional responses among the variants, despite similarly high pulmonary viral RNA load.

Specifically, we demonstrate that while all variants achieved high and comparable pulmonary viral loads, the Gamma variant was associated with prominent perivascular and peribronchiolar inflammation, with severe microvascular injury (vasculitis, edema, and cellular diapedesis), whereas viral Spike was detectable within the perivascular and peribronchiolar compartments only in the Delta variant. At the molecular level, this phenotype correlated with the upregulation of alternative complement pathway components and inflammatory mediators, alongside the downregulation of tissue-adhesion genes. Conversely, the Omicron variant exhibited attenuated transcriptional remodeling and milder clinical outcomes. Together, these findings identify variant-associated differences in pulmonary pathology and host transcriptional responses and provide a framework for further investigation of the biological processes associated with differential disease severity.

## 2. Materials and Methods

### 2.1. Animal Model

C57BL/6J transgenic mice expressing the human angiotensin-converting enzyme 2 receptor (hACE2), under the control of the cytokeratin-18 gene promoter (K18-hACE2), were used. These mice were originally obtained from The Jackson Laboratory (USA) and bred at the Immunopharmacology Laboratory of the Institute of Biological Sciences (ICB) at the Federal University of Minas Gerais (UFMG). The experiments were conducted in the animal facility of the Biosafety Level 3 Laboratory (BSL-3) at UFMG, with 12-week-old male and female mice, maintained in a 12-h light/dark cycle, with water and food ad libitum and under specific pathogen-free conditions. The experiments were conducted following the recommendations of the Guide for the Care and Use of Laboratory Animals of the National Council for the Control of Animal Experimentation (CONCEA) and approved by the Ethics Committee on Animal Experimentation of the Federal University of Minas Gerais (UFMG) on September 13, 2021 (protocol no. 191/2021 CEUA/UFMG).

### 2.2. Virus Stocks

The SARS-CoV-2 viral strains used in this study were clinical isolates of the SARS-CoV-2 variants Alpha B.1.1.7, Gamma P.1, Delta B.1.617.2 and Omicron BA.1. Viral stocks were propagated in Vero CCL81 cells in a humidified incubator at 37 °C with 5% CO_2_ and monitored daily for cytopathic effects (CPE) for up to 72 h. Viruses were titrated in Vero CCL81 cells using plaque-forming unit (PFU) assay and were also sequenced, Gisaid (under accession numbers Alpha: EPI_ISL_3155962; Gamma: EPI_ISL_6514207; Delta: EPI_ISL_6500150 and Omicron: EPI_ISL_16514938). Viral aliquots were maintained at −80 °C.

### 2.3. In Vivo Model

K18-hACE2 mice (five males and three females per group) were randomly assigned to five experimental groups: uninfected Control (Mock), Alpha, Gamma, Delta, and Omicron, with eight animals per group. Within each group, the animals were identified by tail markings and numbered from 1 to 8. The first four animals were designated for the pathogenesis assessment, while the remaining four were followed for survival evaluation.

The mice were then anesthetized with a ketamine and xylazine solution administered and inoculated intranasally with the Alpha, Gamma, Delta, or Omicron variants of SARS-CoV-2. Each animal received 3 × 10^3^ PFU of SARS-CoV-2 in a volume of 30 μL diluted in saline, according to the experimental protocol described by Zheng and coauthors (2021) [28]. The control group was subjected to the same experimental procedures (anesthesia followed by aspiration of the diluent medium). This viral inoculum was selected based on preliminary dose-ranging studies conducted for each SARS-CoV-2 variant in the K18-hACE2 mouse model. Various doses were evaluated to identify an inoculum capable of establishing robust viral infection while preventing mortality throughout the observation period. Notably, 3 × 10^3^ PFU/animal was the sole uniform dose that fulfilled these criteria across all four variants (Alpha, Gamma, Delta, and Omicron).

After infection, the live animals were monitored daily until day 14 post-infection, and clinical signs including, hunched posture, piloerection, lethargy, ocular discharge, and changes in body mass were assessed using a semiquantitative clinical scoring system. Each clinical parameter was scored from 0 to 2, where 0 indicated the absence of clinical signs, 1 indicated mild and 2 indicated severe clinical signs. The individual scores were summed to obtain an overall clinical score for each animal. If a loss of body mass exceeding 20% of the initial body weight was detected, the animal was euthanized by anesthetic overdose. On the 5th day after infection, some of the animals (*n* = 4/group) were euthanized for lung tissue collection for molecular analysis.

### 2.4. Histopathological Analysis and Immunohistochemistry

For histopathological scoring, lung fragments were fixed in 10% neutral buffered formalin, embedded in paraffin, sectioned at 3 μm, and stained with hematoxylin and eosin (H&E) and the analyses was performed in sections in a blinded manner according to previous studies [29,30]. Histopathological changes were evaluated microscopically across the entire section, which was surveyed by dividing each slide into four quadrants (a cross) to ensure complete and systematic coverage rather than selecting representative fields; non-infected mice served as controls for comparative analysis. For immunostaining analyses, lung samples were processed and sections of 3 μm were deparaffinized, rehydrated, and subjected to antigen retrieval using TRILOGY™ solution (Cell Marque, Rocklin, CA, USA; 1:100) in two 20 min baths at 94 °C. Endogenous peroxidase was blocked with hydrogen peroxide (H_2_O_2_ 30v) for 15 s, followed by a distilled-water rinse, and slides were incubated were the following primary antibodies: anti-SARS-CoV-2 (Spike, 1:500; Abcam), F4/80 (D2S9R) XP Rabbit mAb (1:200; cat. #70076), Ly-6G (E6Z1T) Rabbit mAb (1:50; cat. #87048), CD4 (D7D27) Rabbit mAb (1:50; cat. #25229), and CD8α (D4W27) XP Rabbit mAb (1:200; cat. #98941), all from Cell Signaling Technology (Danvers, MA, USA). After washing with Tris buffer (two × 5 min), the EnVision™ detection system (Dako, Carpinteria, CA, USA) was applied according to the manufacturer’s instructions. Color development was achieved using 3,3′-diaminobenzidine (DAB; Spring Bioscience, Pleasanton, CA, USA). Slides were counterstained with Mayer’s hematoxylin, dehydrated in graded ethanol, cleared in xylene, and mounted with Erv-Mount (EasyPath Diagnósticos, Indaiatuba, SP, Brazil). Spleen was used as the positive control for identification of F4/80, Ly-6G, CD4 and CD8α positive cells. Negative control consisted of omission of the primary antibodies. One lung section per animal was immunostained for each of the five markers (anti-Spike, F4/80, Ly-6G, CD4, and CD8), amounting to five sections per animal. Because immunolabeling was infrequent and heterogeneously distributed, in some animals only one or two positive cells were present per section, the entire section was screened using the same four-quadrant survey rather than predefined microscopic fields. For each marker, the presence or absence of labeled cells, the absolute number of immunopositive cells, and the anatomical localization were recorded per whole section by a pathologist blinded to group allocation. Per-animal results were then summarized at the group level as the percentage of positive animals per experimental group.

To characterize the immunopathogenesis of SARS-CoV-2 and its alterations in the lung tissue of hACE2-K18 mice promoted by Alpha, Delta, Gamma, and Omicron variants we evaluate the presence of histopathological alterations in the H&E analysis, considering the intensity of the inflammatory process, presence of hyperemia/congestion, edema, vasculitis, diapedesis, hemorrhage, thrombosis, hemosiderin, necrosis, emphysema, and atelectasis. Each parameter was scored on an ordinal 0–4 scale, (0 = absent; 1 = mild, <10% of the section; 2 = moderate, 10–50%; 3 = intense, 50–75%; 4 = very intense, >75%) based on the extent of involvement across the whole section. Statistical analyses were performed using R software (version 3.4.2), with the ggplot2 (v4.0.1) and gtsummary (v2.6.1) packages. The significance level was set at *p* < 0.05. Because the histopathological lesion scores are ordinal, they are presented as the median and interquartile range and compared among groups using the Kruskal–Wallis test rather than one-way ANOVA. To record data from the immunohistochemical analysis, Fisher’s exact test was applied, considering values of *p* < 0.05.

### 2.5. RNA Isolation and Viral Load Detection

Total RNA was extracted from lung samples (30 mg of tissue) using the RNeasy Mini Kit (Qiagen, Hilden, Germany), and RNA concentration was determined using the Qubit RNA HS Assay kit (Thermo Scientific Fischer, Waltham, MA, USA). Samples were normalized prior to molecular analysis for the same amount of total RNA. HPRT expression was assessed by Real-Time RT-PCR as an additional endogenous control to verify comparable amounts of amplifiable RNA across samples. SARS-CoV-2 detection and quantification were performed according to the CDC’s 2019 Novel Coronavirus (2019-nCoV) Real-Time RT-PCR Diagnostic Panel, using the 2019-nCoV RUO (IDT) assay targeting the N1 and N2 regions of the SARS-CoV-2 nucleocapsid (N) gene. The human RNAse P target was included as an endogenous internal control to assess the adequacy of RNA extraction and the presence of amplifiable RNA in the sample. For quantification, the commercially available 2019-nCoV_N Positive Control (IDT), containing a defined number of target copies, was used to generate a standard curve through serial tenfold dilutions ranging from 10^6^ to 10^1^ copies/μL. The standard curve, consisting of known target copy number, was used to estimate the SARS-CoV-2 RNA copy number in each sample based on the corresponding quantification cycle values (*Ct*) and the results were expressed as SARS-CoV-2 genome copies per microliter (copies/μL) of extracted RNA. Negative controls were included to monitor potential contamination. Real-time RT-PCR was performed using the CFX Opus Real-Time PCR system (Bio-Rad Laboratories, Hercules, CA, USA).

### 2.6. RNA Quality Assessment and RNA Sequencing (RNA-seq)

Total RNA isolated from lung tissue was treated with DNase (TURBO DNA-free kit, Thermo Fisher Scientific, Waltham, MA, USA) to prevent contamination by genomic DNA. RNA was quantified using Qubit RNA HS Assay Kit on a Qubit fluorometer (ThermoFisher Scientific) and RNA integrity was assessed by capillary electrophoresis on a 4150 TapeStation System using a High Sensitivity RNA ScreenTape (Agilent Technologies, Santa Clara, CA, USA). For library preparation, the QIAseq Stranded RNA Library Kit (Qiagen, Hilden, Germany) was used, with rRNA and/or globin depletion, following the manufacturer’s instructions and using 100 ng of total RNA from each sample. Samples were indexed using IDT for Illumina DNA/RNA UD Indexes (Illumina, San Diego, CA, USA). The resulting libraries were quantified using QIAseq Library Quant Assay kit (Qiagen, Hilden, Germany) and average size (300 bp) was verified using High-Sensitivity D1000 ScreenTape reagents (Agilent Technologies, Santa Clara, CA, USA). For RNA sequencing, three animals out of the four available per group (*n* = 3/group) were selected based on three predefined criteria: (i) confirmed pulmonary viral replication quantified by RT-qPCR, (ii) optimal total RNA recovery and integrity, and (iii) optimal library yield. RNA-seq was performed using the NextSeq 500/550 High Output v2 kit (150 cycles) (Illumina, San Diego, CA, USA) on a NextSeq 550 platform (Illumina, San Diego, CA, USA).

### 2.7. RNA-seq and Differential Expression Analysis

The raw data obtained from the sequencing passed through an initial quality analysis step. Low-quality bases (Phred below 30) and Illumina adapters were trimmed with the BBDuk program (v39.91). Quality-filtered reads were initially aligned against the ancestral SARS-CoV-2 reference genome (GenBank NC_045512.2) using Bowtie2 (v2.5.5) [31] to enrich viral reads and discard host background. The resulting mapped reads were then used as input for *de novo* assembly with SPAdes (v4.3.0) [32] to reconstruct the viral genomes and confirm lineage identity across experimental groups. After assembling the viral genome, we also mapped the reads from each library to the *Mus musculus* genome (GRCm38—GCF_000001635.27) using the Bowtie2 program and quantified them using the Salmon software (v2.8.0) [33].

Transcript-level abundance estimates were summarized into gene-level count matrices using *tximport* (v1.28.0) using an Ensembl-to-RefSeq mapping table generated via *biomaRt* (v2.66.0) (dataset *mmusculus_gene_ensembl*). Differential gene expression analysis was conducted in R using the DESeq2 package (v1.40.2) [34]. Data were modeled using a negative binomial generalized linear model with the single-factor design formula design = ~ condition, where condition defined the experimental infection status (Control, Alpha, Delta, Gamma, and Omicron), with the uninfected Control group specified as the reference level. Library size factors were calculated to normalize sequencing depth, and gene-wise dispersions were estimated across all samples. Differential expression was assessed individually for each infected condition relative to the uninfected control (i.e., Alpha vs. Control, Delta vs. Control, Gamma vs. Control, and Omicron vs. Control) using the Wald test with independent filtering of low-count genes to optimize power. Raw *p*-values were corrected for multiple testing using the Benjamini–Hochberg False Discovery Rate (FDR) method [35]. Genes fulfilling the thresholds of adjusted *p*-value (*p* − *adj*) < 0.05 and Log2FoldChange > 2.5 were classified as differentially expressed genes (DEGs).

To evaluate transcriptional differences among all four SARS-CoV-2 variants, an intersection analysis of all identified DEGs across conditions was conducted using the UpSetR package (v1.4.0). Binary classification matrices for up- and down-regulated genes were constructed to evaluate multi-group overlap and identify both conserved and variant-exclusive transcriptional responses. Variance-stabilizing transformation (*vst*) was applied for exploratory data visualization, including Principal Component Analysis (plotPCA) and sample distance heatmaps (*pheatmap*) (v1.0.13) [36].

Functional annotation and pathway enrichment analyses were performed using *clusterProfiler* (v4.8.3) [37] and *ReactomePA* (v1.44.0) [38], utilizing *org.Mm.eg.db* (v3.17.0) [39] as the reference murine annotation database. For standard Gene Ontology (GO) over-representation analysis, all expressed genes identified in the RNA-seq dataset served as the background universe. *p*-values were adjusted for multiple comparisons using the Benjamini–Hochberg method with a significance threshold of *p* − *adj* < 0.05.

Differentially expressed genes (*p* − *adj* < 0.05) were first mapped to canonical Gene Ontology Biological Process (GO-BP) terms using AnnotationDbi and GO.db. Specific GO terms were then systematically clustered into overarching pathological categories that matched the histological scoring criteria evaluated in tissue sections, utilizing a curated keyword-matching algorithm: (i) *Thrombosis/Coagulation* (terms containing *platelet*, *hemostasis*, *clot*, *fibrinolysis*, *coagulation*); (ii) *Hemorrhage* (terms containing *hemorrhage*, *bleeding*, *blood loss*); (iii) *Vasculitis* (terms containing *vasculitis*, *vascular*, *endothelium*, *angiogenesis*, *blood vessel development*); (iv) *Inflammatory infiltrate* (terms containing *inflammatory*, *leukocyte*, *neutrophil*, *macrophage*, *chemotaxis*, *migration*, *infiltration*, *immune response*, *cytokine*, *diapedesis*); (v) *Necrosis/Cell death* (terms containing *necrosis*, *necrotic*, *cell death*, *apoptosis*); (vi) *Edema/Permeability* (terms containing *edema*, *fluid*, *permeability*); (vii) *Hyperemia/Congestion* (terms containing *hyperemia*, *congestion*, *blood circulation*, *vasodilation*, *blood flow*); (viii) *Emphysema/Atelectasis* (terms containing *lung*, *respiratory*, *alveolar*, *emphysema*, *atelectasis*, *gas exchange*); and (ix) *Hemosiderin/Iron ion* (terms containing *hemosiderin*, *iron ion*, *erythrocyte homeostasis*). For each pathological domain, the top 10 genes displaying the highest mean absolute Log_2_FoldChange across variants were selected and visualized using *circlize* (v0.4.15) [40] and *ComplexHeatmap* (v2.16.0) [41].

The complete differential expression tables, effect sizes (Log2FoldChange), and adjusted *p*-values are reported in Supplementary Table S4. The sequencing raw data are deposited in the NCBI SRA under BioProject PRJNA1398918.

## 3. Results

### 3.1. Mouse Infection Model

**Figure 1:**
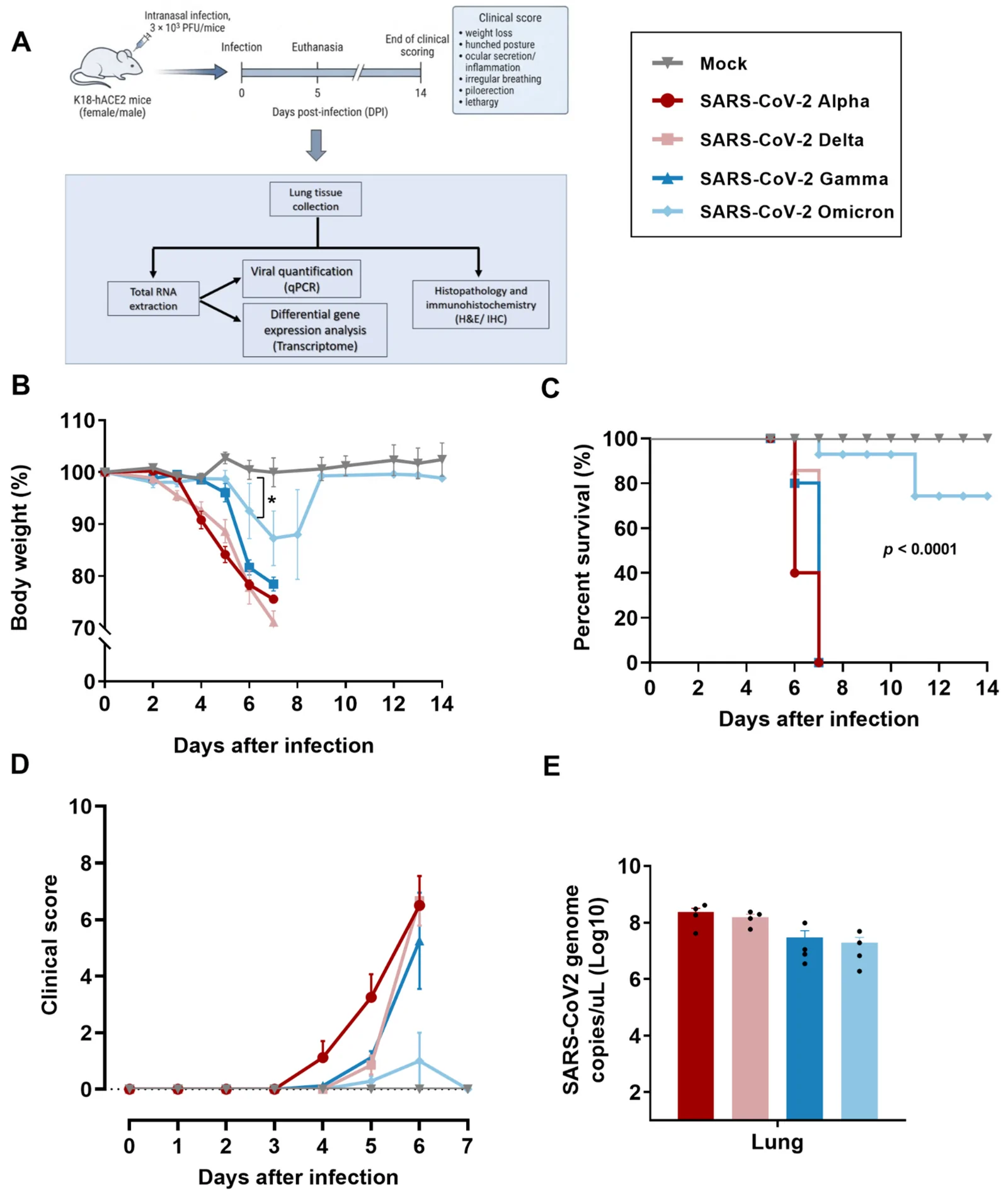
Clinical signs and survival curve of SARS-CoV-2 variants in K18-hACE2 mice model. K18-hACE2 mice infected with SARS-CoV-2 variants Alpha B.1.1.7, Gamma P.1, Delta B.1.617.2 or Omicron BA.1 with (3 × 10^3^ PFU/animal). (**A**) Schematic representation of experimental strategy of K18-hACE2 mice infection showing that 8 animals were infected; 4 euthanized to evaluate pathological and molecular changes and 4 animals followed for 14 days to evaluate survival. (**B**) To body weight evaluation a Mixed-Effects Analysis was performed (*n* = 8/group). The (*) symbol indicates a statiscally significant difference (*p* value < 0.005), (**C**) percentage of survival (*n* = 8/group). Overall survival was estimated using Kaplan-Meier, and statistical differences among groups were determined by the Log-rank (Mantel–Cox) test (*p* < 0.0001) and (**D**) clinical signs of K18-hACE2 mice assessed daily after infection (*n* = 8/group). The clinical signs in the mice were classified as follows: 0 = no clinical signs; 1 = mild signs or 2 = severe signs. (**E**) Viral RNA load of SARS-CoV-2 virus was quantified by qPCR (*n* = 4/group) on the 5th day after infection, with half of the animals in each group euthanized for lung tissue collection. Results were expressed in all subpanels as Mean ± SEM for the number of independent experiments. GraphPad Prism 9.0 (GraphPad Software, Inc, San Diego, CA, USA) was used to analyze data using different tests as appropriate.

To simulate the clinical manifestations of COVID-19 in patients infected with major SARS-CoV-2 variants and to assess the pathology at the primary site of disease, K18-hACE2 mice were infected intranasally with the Alpha, Gamma, Delta, or Omicron variants (3 × 10^3^ PFU/animal; *n* = 8 per group) (Figure 1A). Body weight changes, clinical symptoms, and survival were monitored daily. A subset of animals was euthanized on day 5 post-infection (four animals per group), while disease progression in the remaining animals was assessed over a 14-day period (Figure 1A).

As shown in Figure 1, SARS-CoV-2 infection induced body weight loss in all infected animals starting on day 3 post-infection, with the exception of the control group (Figure 1B). This progressive loss of body mass was accompanied by severe acute clinical signs, including piloerection, hunched posture, ocular discharge, lethargy, and even respiratory distress. Mice infected with the Alpha variant exhibited the earliest clinical symptoms, beginning on day 3 post-infection. Moreover, animals infected with the Alpha, Gamma, and Delta variants displayed the worst clinical scores. Infection with the Omicron variant resulted in milder and delayed symptoms (Figure 1D), as well as reduced body mass loss (Figure 1B); affected animals were able to recover their initial body weight by the end of the 14-day observation period (Figure 1B).

Regarding survival, all animals infected with the Alpha, Gamma, and Delta variants exhibited higher lethality, with survival lasting up to seven days post-infection, highlighting the severity of disease caused by these variants (Figure 1C) despite no deaths detected with Omicron infected animals.

To assess viral RNA load and potential pathological damage in lung tissue, lungs from infected K18-hACE2 mice were collected on day 5 post-infection (four mice per group). High levels of viral RNA were detected in lung tissue, ranging from 10^7^ to 10^8^ copies/µL indicating a high viral RNA; however, no significant differences in viral RNA levels were observed among animals infected with the different variants (Figure 1E).

### 3.2. Variant-Dependent Pulmonary Tropism and Immune Cell Recruitment in SARS-CoV-2-Infected hACE2-K18 Mice

**Figure 2:**
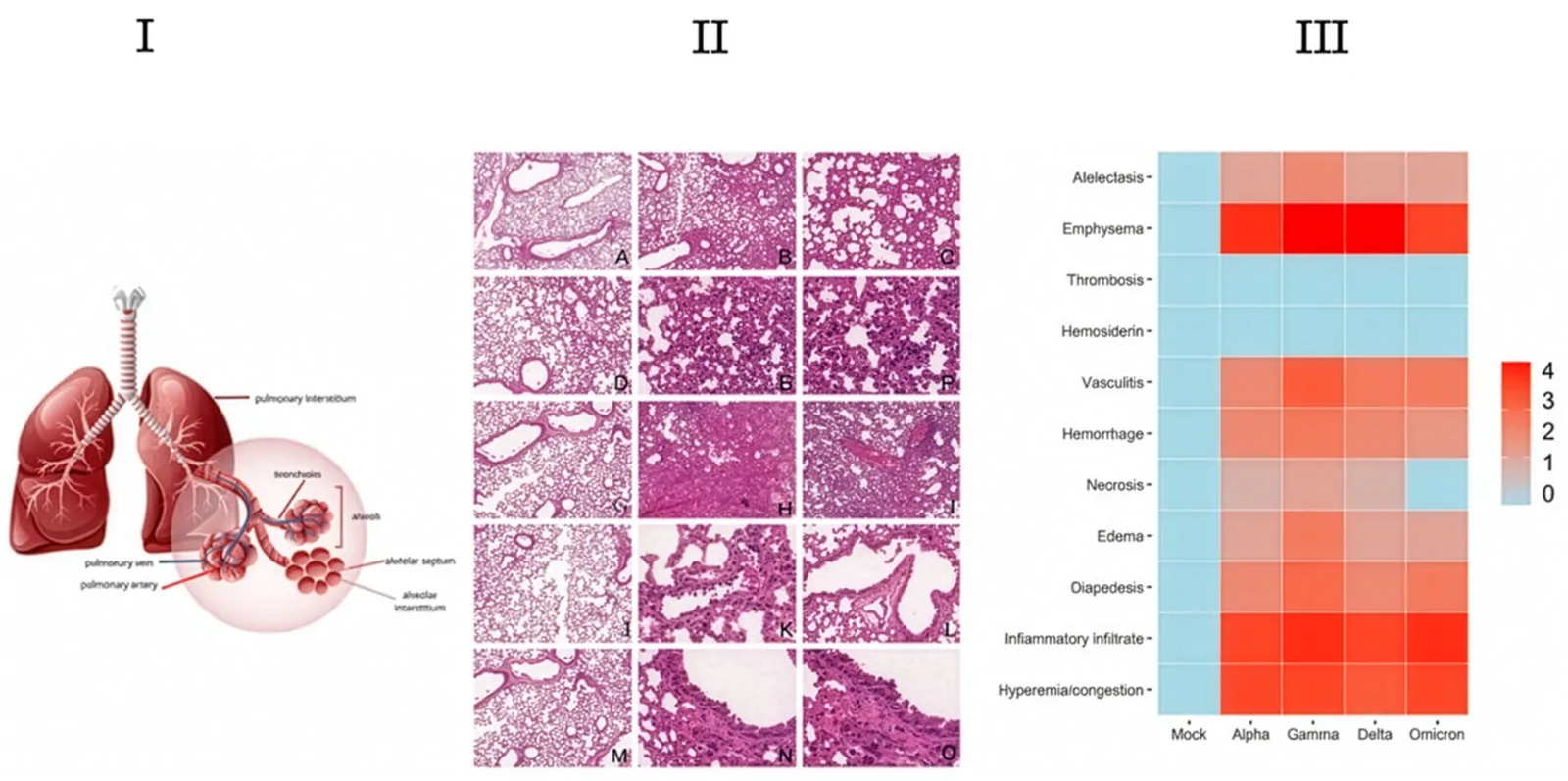
Histological findings in control group and Gamma-infected variant in K18-hACE2 mice model. (**I**) Schematic representation of the murine pulmonary architecture and anatomical compartments analyzed. (**II**) Representative lung histological sections from control animals (**A**,**D**,**G**,**J**,**M**; 100×) and animals infected with the Gamma variant (**B**,**C**,**E**,**F**,**H**,**I**,**K**,**L**,**N**,**O**). Histological findings include intense inflammatory infiltrate, emphysema, and atelectasis (**B**,**C**); alveolar hemorrhage (**E**,**F**); exudative edema (**H**,**I**); inflammatory cell diapedesis and vasculitis (**K**,**L**); and focal necrosis (**N**,**O**). Panels (**C**,**F**,**H**,**K**,**O**) show these findings at 400×, while the corresponding paired panels show them at lower magnification. (**III**) Heatmap showing histopathological changes across the experimental groups. Colours indicate histopathological scores, as shown by the colour scale: 0 = absent; 1 = mild (<10% of the section); 2 = moderate, (10–50%); 3 = intense (>50–75%); 4 = very intense (>75%).

**Figure 3:**
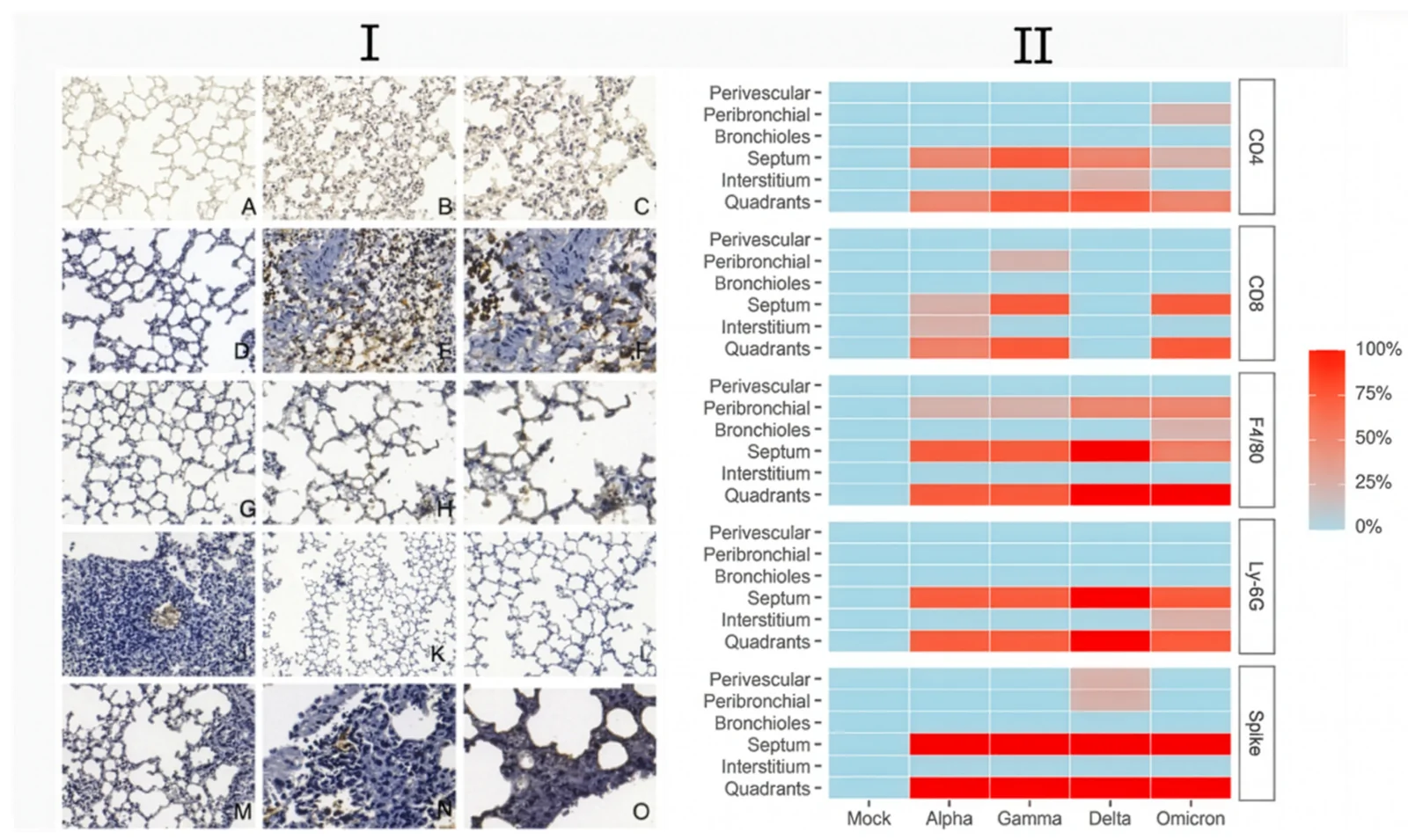
Compartment-specific pulmonary distribution of SARS-CoV-2 Spike protein and immune cell infiltration per variant of SARS-CoV-2 in K18-hACE2. (**I**) Representative photomicrographs of lung tissue sections from K18-hACE2 mice infected with SARS-CoV-2 variants (Alpha, Gamma, Delta, and Omicron) or non-infected controls evaluated five days post-infection. Sections show immunohistochemical staining for the following markers: CD4^+^ T cells (panels **A**–**C**), CD8^+^ T cells (panels **D**–**F**), macrophages (F4/80^+^, panels **G**–**I**), neutrophils (Ly-6G^+^, panels **J**–**L**), and SARS-CoV-2 Spike protein (panels **M**–**O**). Specific panels demonstrate (**C**) septal CD4^+^ recruitment in Gamma; (**E**,**F**) septal and lymphoid aggregate CD8^+^ infiltration in Gamma; (**H**,**I**) robust septal and interstitial F4/80^+^ macrophages in Delta; (**K**,**L**) extensive septal Ly-6G^+^ neutrophils in Delta; and (**N**,**O**) prominent septal Spike expression in Gamma and Delta. (**II**) Heatmaps summarizing the percentage of animals positive for each marker across the anatomical categories analyzed. Colours indicate the percentage of positive animals in each group (*n* = 4), as shown by the colour scale. Corresponding numerical values are provided in Supplementary Table S1.

For the immunohistochemical and compartmental analysis, five anatomical lung regions were evaluated—the alveolar septum, the pulmonary interstitium, the bronchiolar region, the peribronchial region and the perivascular space, as represented schematically in Figure 2A. In addition, positivity detected anywhere within the four-quadrant survey of the whole section was recorded separately as “Quadrants”. Consequently, six categories are reported in Supplementary Table S1 and in Figure 3B. The distribution of the Spike viral protein and the immune cell markers across these compartments is presented below (Figure 3A,B and Supplementary Table S1).

Histopathological examination of lung sections stained with H&E revealed marked structural disruption and inflammatory infiltration following SARS-CoV-2 infection (Figure 2A–O). Lung tissues from infected groups exhibited prominent septal thickening, alveolar collapse (atelectasis), alveolar enlargement consistent with emphysema, and focal areas of vascular damage with peri-bronchovascular cellular cuffs compared to uninfected controls (Figure 2B).

Semiquantitative grading of these histopathological alterations (0 = absent; 1 = mild, <10% of the section; 2 = moderate, 10–50%; 3 = intense, 50–75%; 4 = very intense, >75%) are summarized as the median and interquartile range in Figure 2C and Supplementary Table S2. Hemosiderin and thrombosis were absent in all groups, and necrosis did not differ significantly between groups. The control group showed no histopathological alterations (median 0 for all parameters), whereas all SARS-CoV-2-infected groups developed pulmonary lesions; the Kruskal–Wallis test including the control group was significant for hyperemia/congestion, inflammatory infiltrate, leukocyte diapedesis, edema, hemorrhage, vasculitis, emphysema, and atelectasis (*p* < 0.05), consistent with infection-induced pulmonary damage. When the control group was excluded, however, none of the histopathological parameters differed significantly among the four viral variants (Kruskal–Wallis, *p* > 0.05 for all parameters), indicating that Alpha, Gamma, Delta, and Omicron BA.1 produced a broadly comparable spectrum of pulmonary lesions by semiquantitative scoring. Although Gamma showed numerically higher median scores for some vascular parameters, such as vasculitis, these inter-variant differences did not reach statistical significance (Figure 2B,C, Supplementary Table S2).

Immunohistochemical analysis demonstrated widespread pulmonary expression of the SARS-CoV-2 Spike protein across all infected groups, in agreement with the comparable viral loads observed among variants (Figure 3I, M–O; Figure 3II, Supplementary Table S1). Among all evaluated anatomical compartments, the alveolar septum and lung quadrants showed robust viral Spike immunoreactivity across all variants (100%, *p* = 0.001). However, the Delta variant exhibited a distinctive tissue penetration pattern, being the only lineage detected within both perivascular (25%) and peribronchial (25%) compartments (Figure 3II, Supplementary Table S1). This perivascular localization aligns with the vascular damage and peribronchiolar inflammation previously observed for this variant (Figure 2III).

Infiltration of innate and adaptive immune cell subsets also displayed variant- and compartment-specific profiles (Figure 3I, A–L; Figure 3II). Macrophages (F4/80^+^) and neutrophils (Ly-6G^+^) constituted the predominant inflammatory populations within alveolar septa and tissue quadrants across all infected groups, with Delta eliciting the highest septal infiltration frequency for both cell types (100%). In contrast, T-cell recruitment was differentially modulated. The CD4^+^ T cells were most prominently detected in the septa of Gamma-infected lungs (75%), whereas CD8^+^ T-cell infiltration was frequent in Gamma and Omicron (75%) but absent in Delta (0%) (Figure 3B, Supplementary Table S1). Together, these findings indicate that while all variants effectively infect the alveolar epithelium, Delta and Gamma drive heightened localized inflammatory responses characterized by distinct myeloid and lymphoid infiltration dynamics.

Hierarchical clustering of the semiquantitative histopathological and immunohistochemical scores (Supplementary Figures S1 and S2) did not segregate animals according to viral lineage: samples from all four variants distributed across the same clusters. In both analyses, a single Gamma-infected animal (A4) branched separately. This animal showed an unusually intense inflammatory infiltrate that effaced the alveolar architecture, together with focal cytoplasmic Spike immunoreactivity restricted to one quadrant of the section. This pattern is therefore consistent with inter-individual variability rather than with a Gamma-specific histopathological signature, in line with the absence of statistically significant differences among variants in the scored histopathological parameters (Supplementary Table S2).

To explore similarities in the anatomical distribution of immunoreactivity, semiquantitative profiles were compared across animal–marker combinations, revealing four major clusters (Supplementary Figure S2). All five marker profiles from Gamma-infected animal A4 (F4/80, Ly-6G, CD8, Spike, and CD4) formed a separate cluster, which joined the remaining profiles at the highest linkage distance. This indicates that the anatomical distribution of immunoreactivity in this animal differed from that observed in the other animals. The remaining profiles formed three clusters containing combinations from all four viral variants. Cluster 2 comprised A3 Alpha (CD8), A4 Omicron (Ly-6G), A3 Delta (CD4), A3 Omicron (F4/80), A3 Gamma (CD8), A1 Omicron (F4/80), and A2 Omicron (CD4). Cluster 3 contained predominantly F4/80, Ly-6G, and Spike profiles, together with selected CD4 and CD8 profiles. Cluster 4 was the largest and contained profiles for all five markers, including many combinations from animals A1 and A2. Thus, the clustering did not show consistent segregation by viral variant. Histological examination of A4 Gamma showed intense inflammatory infiltration with marked disruption of the alveolar architecture. Spike immunoreactivity was focal and cytoplasmic, involving cells morphologically consistent with pneumocytes along the alveolar septa within a single quadrant of the section. These observations describe pronounced tissue injury accompanied by focal detectable viral antigen, rather than demonstrating extensive viral dissemination. Overall, the distinct clustering of A4 Gamma is consistent with inter-individual variability rather than with a Gamma-specific immunohistochemical signature.

### 3.3. Viral RNA Expression

RNA sequencing generated 15 high-quality libraries comprising both control and SARS-CoV-2-infected samples (Supplementary Table S3). These included libraries infected with different VOCs (*n* = 12; three libraries per VOC) and uninfected control libraries (*n* = 3). Viral reads were detected in all infected libraries, and near-complete viral genomes were successfully assembled for each condition (Supplementary Table S3).

### 3.4. Conserved Interferon Responses and Variant-Specific Transcriptional Modulation Across SARS-CoV-2 VOCs

**Figure 4:**
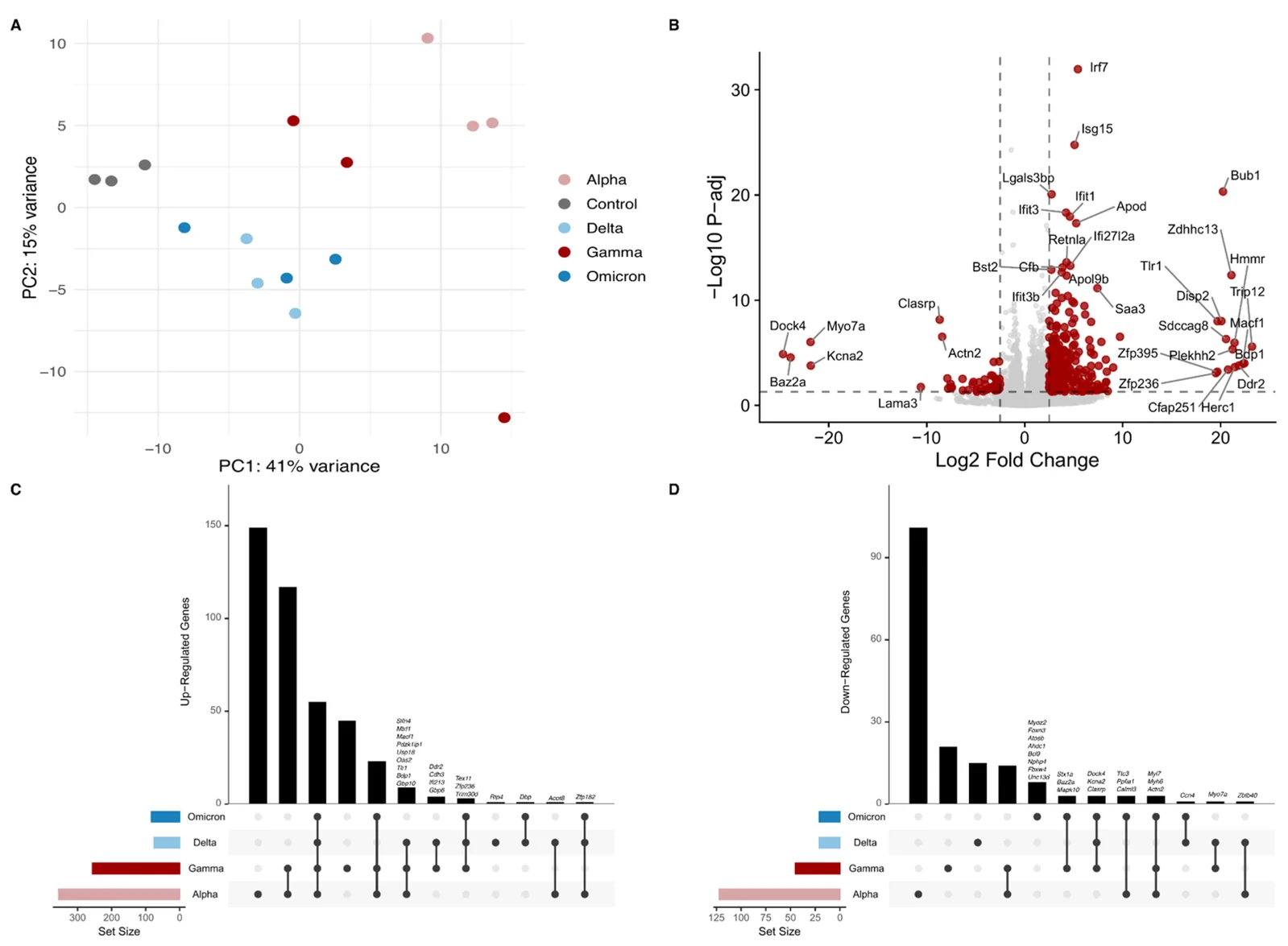
Transcriptomic profiling and statistical landscape of differential host gene expression across SARS-CoV-2 VOCs. (**A**) Principal Component Analysis (PCA) of murine lung transcriptomes based on variance-stabilizing transformed (VST) normalized counts across experimental conditions (Alpha, Gamma, Delta, Omicron, and uninfected Control; *n* = 3 biological replicates per group). Percentages along axes indicate the proportion of explained variance for PC1 (41%) and PC2 (15%). (**B**) Differential gene expression between uninfected controls and the Gamma variant. Statistical significance was calculated using the two-tailed Wald test in DESeq2, and *p*-values were adjusted for multiple testing via the Benjamini–Hochberg false discovery rate (FDR) procedure. The horizontal dashed line denotes the statistical significance cutoff (−*log*_10_*p* − *adj* = 1.30, equivalent to *p* − *adj* = 0.05); vertical dashed lines denote biological effect-size thresholds (Log2FoldChange > 2.5). Transcripts fulfilling both criteria (*p* − *adj* < 0.05 and Log2FoldChange > 2.5) are highlighted in red. (**C**,**D**) UpSet plots illustrating the combinatorial intersections of significantly (**C**) upregulated and (**D**) downregulated genes across SARS-CoV-2 variants. Only genes meeting the predefined statistical criteria (*p* − *adj* < 0.05, FDR corrected, and Log2FoldChange > 2.5) in each independent contrast against uninfected controls were included. Vertical bars indicate the number of statistically significant genes unique to or shared across overlapping infection sets. Annotated gene names are shown for subsets containing fewer than 10 genes. Complete gene lists are available in Supplementary Table S4.

To further investigate global transcriptional differences among conditions, we performed a PCA and showed that samples infected with the Gamma and Alpha variants formed well-defined and distinct clusters, clearly separated from both the control group and the other infected conditions. In contrast, samples infected with the Delta and Omicron VOCs clustered closer to each other, indicating a more similar transcriptional response (Figure 4A).

The differential expression analysis of all four infection conditions highlighted a robust antiviral response. As an example, the Gamma (Figure 4B) showed upregulation of classic Interferon-Stimulated Genes (ISGs), including *Irf7*, *Isg15*, *Ifit1*, *Ifit3*, and *Bst2*. Conversely, genes associated with structural integrity and adhesion, such as *Lama3* and *Ddr2*, were significantly downregulated.

In the other conditions, a substantial number of genes were also shared across different variants. For instance, in Alpha (Supplementary Figure S3A), ISGs such as *Irf7*, *Isg15*, *Ifit1*, and *Ifit3* were markedly expressed, a pattern similarly observed in Gamma infection. In contrast, infection with the Delta resulted in a comparatively distinct transcriptional profile, characterized by a smaller number of significant differentially expressed genes. Although classical ISGs remained upregulated, additional genes associated with vesicle trafficking and transcriptional regulation, such as *Baz2a* and *Foxn3,* were modulated in this condition (Supplementary Figure S3C). The Omicron exhibited a transcriptional pattern that partially overlapped with those of the other VOCs; however, it was characterized by overall lower statistical significance and reduced magnitudes of fold change across differentially expressed genes (Supplementary Figure S3D).

Considering that SARS-CoV-2 VOCs shared a substantial number of differentially expressed genes (DEGs), we performed an intersection analysis to identify core and variant-specific gene signatures, taking into account both upregulated and downregulated genes. We identified a core set of 117 upregulated genes shared by all four variants (Figure 4C), representing a conserved antiviral host response. Notably, the largest intersection of upregulated genes (*n* = 149) was shared among the Alpha, Gamma, and Delta variants, but was absent in Omicron infection. A similar pattern was observed for downregulated genes (Figure 4D), in which 101 genes were commonly suppressed by Alpha, Gamma, and Delta, but not by Omicron.

Specifically for Alpha, a distinct downregulation of genes associated with adaptive immune responses and surface receptor signaling was observed. Notable examples include *Cd79a*, which is involved in B cell receptor signaling, and *Vpreb3*, a component of the immunoglobulin complex, suggesting a potential early negative impact on B lymphocyte differentiation or activation under this condition. In addition, genes related to structural organization and synaptic regulation, such as *Cttnbp2* and *Srpk3*, were also exclusively repressed.

The transcriptomic response to the Gamma variant revealed a distinct signature marked by the modulation of humoral innate immune-related transcripts. Among the uniquely upregulated genes in this condition, *Cfb* stood out as a key component of the alternative complement pathway. While functional cascade activation was not directly quantified, this marked transcriptional induction suggests a potential link between alternative complement pathway signaling and the heightened pulmonary inflammation observed during Gamma infection. Additionally, the induction of *Retnla*, a secreted protein linked to intense inflammatory processes and immune cell recruitment, was observed. In contrast, analysis of downregulated transcripts revealed changes associated with altered tissue integrity and cell adhesion. Genes involved in extracellular matrix maintenance and basement membrane organization, such as *Lama3*, were significantly repressed, which is consistent with impaired extracellular matrix organization and the extensive pulmonary architectural damage observed histologically.

For Delta, among the genes exclusively upregulated during Delta infection, *Rtp4* was identified. Omicron displayed a unique gene repression signature distinct from the other variants. We identified exclusive downregulation of *Unc13d*, a gene critical for lytic granule exocytosis and the regulation of NK cell and mast cell degranulation, functions essential for effective cellular cytotoxicity. Table S5 contains the list of all genes shared among and exclusive to each infection condition, including both upregulated and downregulated genes.

### 3.5. Pathological Functional Enrichment Analysis

**Figure 5:**
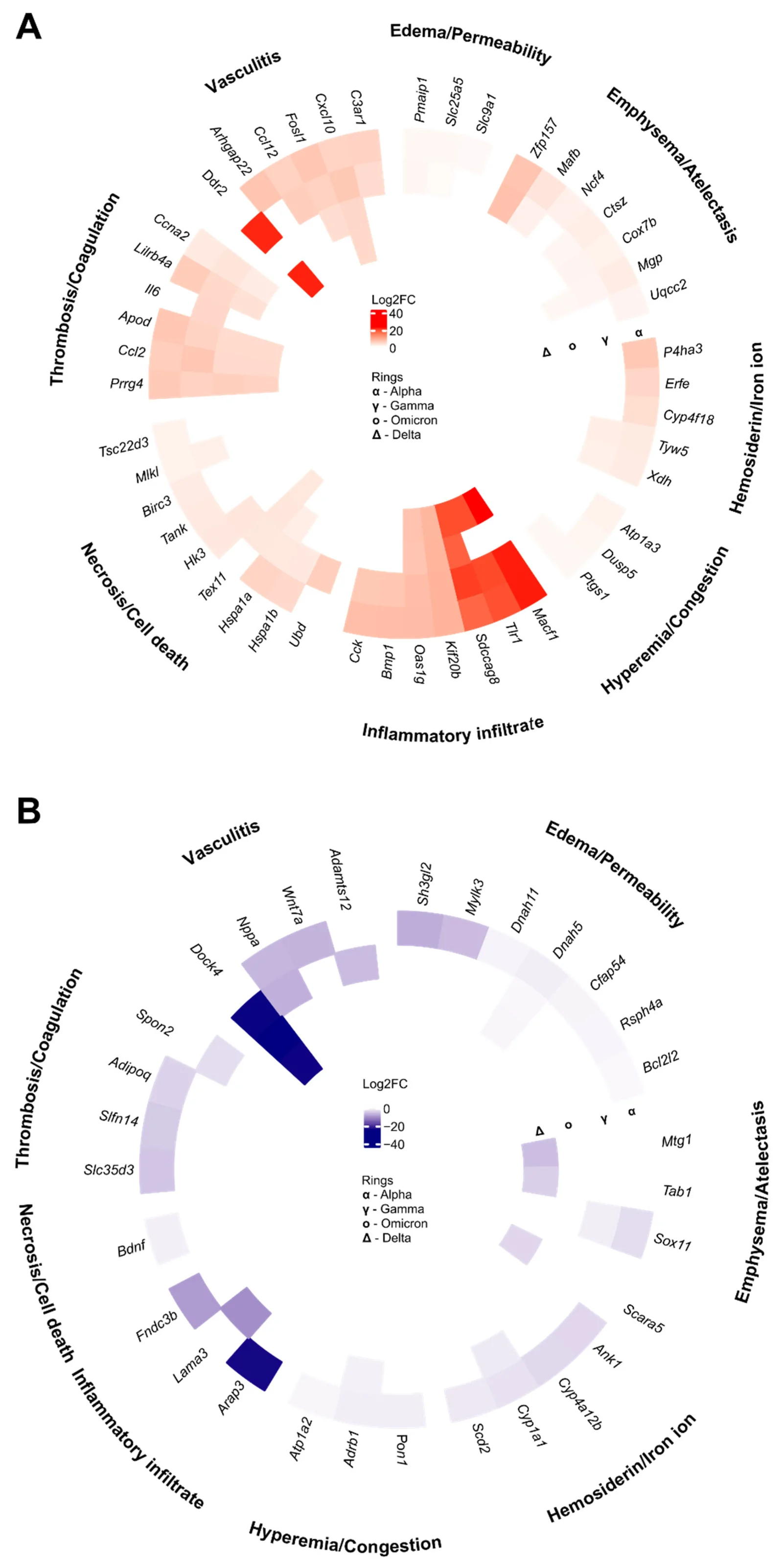
Landscape of differential gene expression classified across histological pathological domains in response to SARS-CoV-2 VOCs. Canonical Gene Ontology Biological Process terms associated with significant DEGs (*p* − *adj* < 0.05) were mapped to targeted pathological categories corresponding directly to the lung lesions scored histologically (Figure 2B,C) and cellular infiltration evaluated by immunohistochemistry (Figure 4B). (**A**) Circos plot displaying the top 10 upregulated genes per pathological category, ranked by mean absolute *Log*_2_*FoldChange* across variants. (**B**) Circos plot displaying the top 10 downregulated genes per pathological category. The concentric heatmap rings represent the averageLog2foldChange for each VOC compared to controls, ordered from inside to outside (A: Alpha, G: Gamma, O: Omicron, D: Delta). Pathological sector labels represent biologically interpreted domains curated from underlying Gene Ontology annotations to reflect tissue pathology.

To further dissect the biological implications of the variant-specific gene expression signatures and bridge the molecular findings with tissue injury, we mapped differentially expressed genes to canonical Gene Ontology Biological Process terms, which were subsequently categorized into targeted pathological modules corresponding directly to the histopathological lesions (Figure 2B,C) and immunohistochemical profiles (Figure 4B) observed in infected lungs (Figure 5). A distinct polarization of pathological signatures among variants was observed. All four VOCs were strongly associated with the upregulation of genes clustered under inflammatory infiltrate and vasculitis-related processes.

Genes classified as upregulated were predominantly associated with inflammatory responses, vascular alterations, and cell death pathways. Pathways related to inflammatory infiltrate and hyperemia exhibited the strongest transcriptional activation, especially in samples infected with the Alpha, Gamma and Omicron variants. Genes such as *Mcl1*, *Tlr1*, and *Sdcag8* showed increased expression levels. Genes involved in vasculitis and coagulation, including *Ccl12* and *Fosl1*, were also significantly upregulated, with the Alpha and Gamma variants displaying *Ccl12* expression compared to Delta and Omicron. Additionally, genes associated with necrosis and cell death, such as *Birc3* and *Hspa1a/b*, were consistently upregulated across variants; however, the magnitude of expression was lower in Omicron-infected samples, suggesting a reduced degree of cellular stress and tissue damage (Figure 5A).

In contrast, downregulated genes were mainly associated with vascular homeostasis, tissue remodeling, and specific subsets of inflammatory pathways. Genes linked to vasculitis and edema, including *Nppa* and *Wnt7a*, were suppressed, especially in the Alpha variant. Interestingly, despite the overall upregulation of inflammatory pathways, certain inflammatory-related genes such as *Arap3* and *Lama3* were significantly downregulated, especially in Alpha and Gamma variants, highlighting a complex and variant-specific regulation of immune responses. Overall, these findings demonstrate that earlier variants of concern, particularly Alpha and Gamma, induce more extensive transcriptomic remodeling, characterized by strong induction of inflammatory and cell death-related genes alongside suppression of genes involved in vascular integrity. In contrast, the Omicron and Delta variants present a more attenuated transcriptional profile, with fewer genes reaching extreme levels of differential expression, which may be indicative of reduced pathogenicity and tissue damage (Figure 5B).

## 4. Discussion

The emergence of SARS-CoV-2 VOCs has reshaped the trajectory of the COVID-19 pandemic. Our study provides a comprehensive comparative analysis of the pathogenesis of Alpha, Gamma, Delta, and Omicron variants in the K18-hACE2 transgenic mouse model. We observed that while all variants achieved comparable pulmonary viral loads, the clinical and pathological outcomes were mainly divergent. Alpha, Gamma, and Delta variants induced 100% mortality within seven days post-infection, whereas the Omicron variant elicited an attenuated, self-limiting phenotype. This dissociation between viral burden and clinical severity Gamma showed numerically higher median scores for vasculitis, alveolar edema and leukocyte diapedesis, although these inter-variant differences did not reach statistical significance; hierarchical clustering did not segregate animals by lineage, and a single Gamma animal (A4) branched separately as an outlier. The clinical phenotype observed for the Alpha variant in our model mirrors robust epidemiological data. The Alpha variant was associated with a higher risk of hospital admission and increased mortality rates in the UK and globally [4,42]. In our K18-hACE2 model, Alpha exhibited rapid disease progression, setting high levels of mortality that were subsequently matched by the Gamma and Delta variants. While Alpha circulation remained lower in Brazil, our data support the hypothesis that it possesses an intrinsic virulence profile that exacerbates respiratory pathology, prefiguring the outcomes observed later with the Gamma variant. The high lethality of Gamma observed in our study data correlates with the mortality rates documented during the pre-vaccination period in Brazil. Our results are consistent with published studies [10,24], which reported similarly high virulence profiles for Gamma and Delta in K18-hACE2 mice, contrasting with the reduced pathogenicity of Omicron [31,35,43].

A hallmark of our study is the microvascular pathology triggered by the Gamma variant. Histopathological scoring revealed that Gamma infection elicited vasculitis, alveolar edema, and leukocyte diapedesis, distinguishing it as the lineage with the most aggressive vascular phenotype. While the Delta variant was uniquely detected within perivascular and peribronchiolar compartments by IHC, Gamma drove heightened vascular damage alongside pronounced septal CD4^+^ T-cell recruitment. Hierarchical clustering analysis supported this divergence, isolating Gamma samples as independent outliers driven by hyper-inflammatory and vascular remodeling. At the molecular level, this pronounced vascular injury is supported by the significant downregulation of *Lama3*, a transcript encoding a laminin subunit for extracellular matrix organization and basement membrane structural integrity. Although our transcriptomic data do not prove that *Lama3* repression directly drives vascular barrier disruption, its downregulation is consistent with impaired tissue architecture and compromised extracellular matrix stability [44]. In the context of viral pneumonitis, such basement membrane destabilization may act synergistically with inflammatory cellular infiltration, reinforcing the tissue disorganization observed in our histological scoring. Alongside *Lama3*, it was also observed the induction of *Cfb*, a component of the alternative complement pathway involved in airway hyperresponsiveness and vascular inflammation [45]. While the transcriptional upregulation of *Cfb* does not directly establish functional enzymatic activation or the cleavage of downstream anaphylatoxins, hyperactivation of the complement cascade, particularly the C5a–C5aR1 signaling axis, is widely documented in the literature as an important driver of endothelial damage and hyperinflammation in severe COVID-19 [45].

The histological profile of animal A4 suggests an association between focal Spike immunoreactivity and pronounced inflammatory damage. Despite the focal distribution of detectable Spike staining, this animal showed extensive inflammatory infiltration and disruption of the alveolar architecture, raising the possibility that the host inflammatory response contributed to the severity of tissue injury. However, these observations do not establish the temporal or causal relationship between viral antigen expression and inflammation, nor do they directly demonstrate the mechanisms underlying the damage. As this pattern was observed in a single outlier animal, it is best interpreted as inter-individual variability rather than as a characteristic response to the Gamma lineage.

Regarding the transcriptional landscape, our analysis identified a conserved antiviral core of 117 upregulated genes across all variants, including interferon-stimulated genes (ISGs) such as *Ifit1* and *Isg15*. However, the divergence in lethality is dictated by the secondary inflammatory remodeling. While Omicron elicits a self-limiting response, the Gamma and Alpha variants trigger extensive transcriptomic dysregulation characterized by an exaggerated inflammatory signature, including genes like *Retnla*. Furthermore, recent evidence indicates that severe SARS-CoV-2 infection drives significant epithelial alterations, such as the upregulation of *EpCAM* in respiratory epithelial cells, which is linked to a hypercellular profile during severe disease [46]. These findings are consistent with the observed transcriptomic remodeling and suggest that variants such as Gamma reprogram the epithelium toward dysregulated proliferation and defective tissue repair, compromising epithelial homeostasis. Consequently, particularly during Gamma and Alpha infections, antiviral immune responses appear to progress from effective viral containment to pathological inflammation. This aligns with findings published previously [4], which described that impaired or dysregulated type I interferon signaling in severe COVID-19 patients leads to progressive lung damage rather than viral resolution [4].

Conversely, the Omicron variant exhibited an attenuated transcriptional profile. Our data showed that while Omicron replicates effectively, it lacks the extreme upregulation of genes associated with vascular remodeling and severe cell death observed in Gamma or Alpha-infected lungs. This finding is consistent with the recent literature suggesting that Omicron’s reduced pathogenicity is linked to altered TMPRSS2 usage. Unlike previous VOCs such as Alpha and Delta, which efficiently utilize TMPRSS2 for cell-surface entry, Omicron exhibits significantly reduced spike cleavage efficiency by this protease. Consequently, Omicron relies predominantly on the endosomal pathway (cathepsin-dependent entry) for viral uptake. This pathway is functional in the upper respiratory epithelium but is less efficient in the deep alveolar parenchyma, where TMPRSS2 expression is typically high. This shift in entry mechanism restricts Omicron’s efficient replication primarily to the upper airways, thereby limiting deep lung parenchymal involvement and mitigating the severe inflammatory and vascular damage observed with earlier VOCs [47]. Conversely, although Omicron has generally been associated with attenuated disease phenotypes, clinically relevant adverse outcomes have continued to be reported in specific populations, including pregnant women during periods of BA.5/BF.7 circulation [48].

In conclusion, our study shows that Alpha and Gamma VOCs were associated with perivascular and microvascular alterations accompanied by extensive transcriptomic remodeling and by transcriptional signatures suggestive of a vasculitis-complement-inflammatory axis, which we propose as a hypothesis warranting further experimental validation. Gamma displays aggressive perivascular and microvascular pathology that correlates with severe tissue damage and mortality. This study demonstrates the distinct pathogenic mechanisms inherent to each variant and how they modulate the host’s immune environment. By mapping these variant-specific signatures, we provide a molecular framework for understanding the divergence in clinical outcomes observed throughout the pandemic and the varying degrees of immune dysregulation that characterize these infections.

## Data Availability

The raw RNA sequencing data generated in this study have been deposited in the NCBI Short Read Archive (SRA) database under BioProject accession number PRJNA1398918.

## Supplementary Materials

The following supporting information can be downloaded at https://www.mdpi.com/article/10.3390/microorganisms14102400/s1, Table S1: Frequency of animals positive for SARS-CoV-2 Spike protein and for immune cell markers in each pulmonary compartment K18-hACE2 mice five days after infection. Table S2: Semiquantitative scores histopathological alterations in the lungs of K18-hACE2 mice five days after infection. Figure S1: Hierarchical clustering analysis of pulmonary histopathological scores across SARS-CoV-2 variants. Dendrogram illustrates sample grouping based on semiquantitative histological scores across infected animals (A1–A4 denote individual biological replicates within Alpha, Gamma, Delta, and Omicron groups). Hierarchical clustering was performed using Euclidean distance and average linkage, identifying four distinct clusters. Dashed boxes delineate the four identified clusters. Y-axis represents the distance (dissimilarity metric) between sample profiles. Supplementary Figure S2: Hierarchical clustering analysis combining anatomical compartment distribution, antibody markers, and viral variants. Dendrogram representing sample clustering based on the combined semiquantitative immunohistochemical scores of viral Spike protein and immune cell markers (CD4, CD8, F4/80, and Ly-6G) across pulmonary anatomical compartments. Hierarchical clustering was conducted using Euclidean distance and average linkage. Dashed boxes indicate the four defined clusters. The Y-axis represents the distance (dissimilarity metric) between sample profiles. Table S3: Summary of RNA-Seq sequencing metrics, host and viral genome mapping, and viral quantification across experimental groups. Supplementary Figure S3: Transcriptomic profiling and differentially expressed genes (DEGs) in lung tissue across SARS-CoV-2 variants. Table S4: Functional annotation and sharing pattern of up-regulated and down-regulated differentially expressed genes (DEGs) across SARS-CoV-2 variants. Table S5: Functional annotation, Gene Ontology terms, and sharing patterns of differentially expressed genes (DEGs) across SARS-CoV-2 variants (Alpha, Gamma, Delta, and Omicron).
