# Supplementary material for: SARS-CoV-2 Gamma Variant In Vivo Model Demonstrates Severe Pathogenicity and Unique Transcriptomic Signatures Compared to Alpha, Delta, and Omicron Variants
Source: Microorganisms. 2026 Oct 20;14(10):2400. doi: 10.3390/microorganisms14102400 (PMC13707728; doi:10.3390/microorganisms14102400)
Supplement: Supplementary file 1 [file microorganisms-14-02400-s001.zip › Supplementary_Figure S1.pdf]

Figure S1

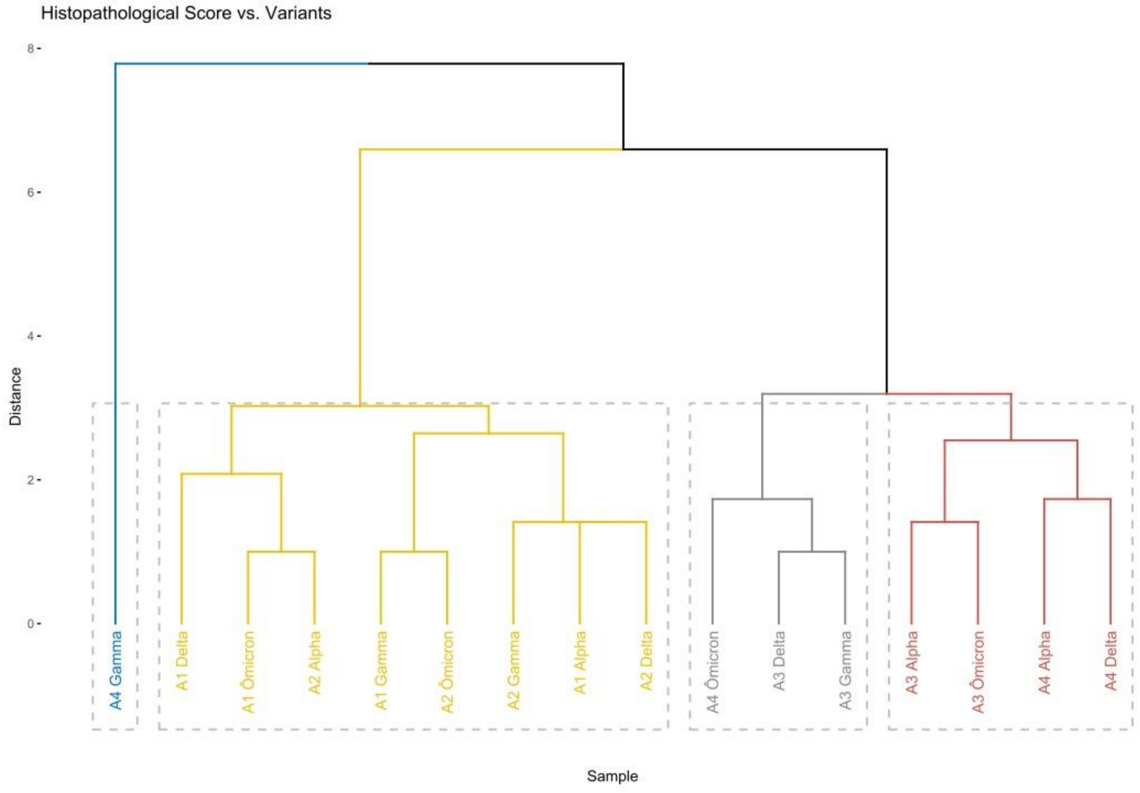
Histopathological Score vs. Variants
Distance
8
6
4
2
0
A4 Gamma
A1 Delta
A1 Ômicron
A2 Alpha
A1 Gamma
A2 Ômicron
A2 Gamma
A1 Alpha
A2 Delta
A4 Ômicron
A3 Delta
A3 Gamma
A3 Alpha
A3 Ômicron
A4 Alpha
A4 Delta
Sample
