# Supplementary material for: SARS-CoV-2 Gamma Variant In Vivo Model Demonstrates Severe Pathogenicity and Unique Transcriptomic Signatures Compared to Alpha, Delta, and Omicron Variants
Source: Microorganisms. 2026 Oct 20;14(10):2400. doi: 10.3390/microorganisms14102400 (PMC13707728; doi:10.3390/microorganisms14102400)
Supplement: Supplementary file 1 [file microorganisms-14-02400-s001.zip › Supplementary_Figure S2.pdf]

Figure S2.

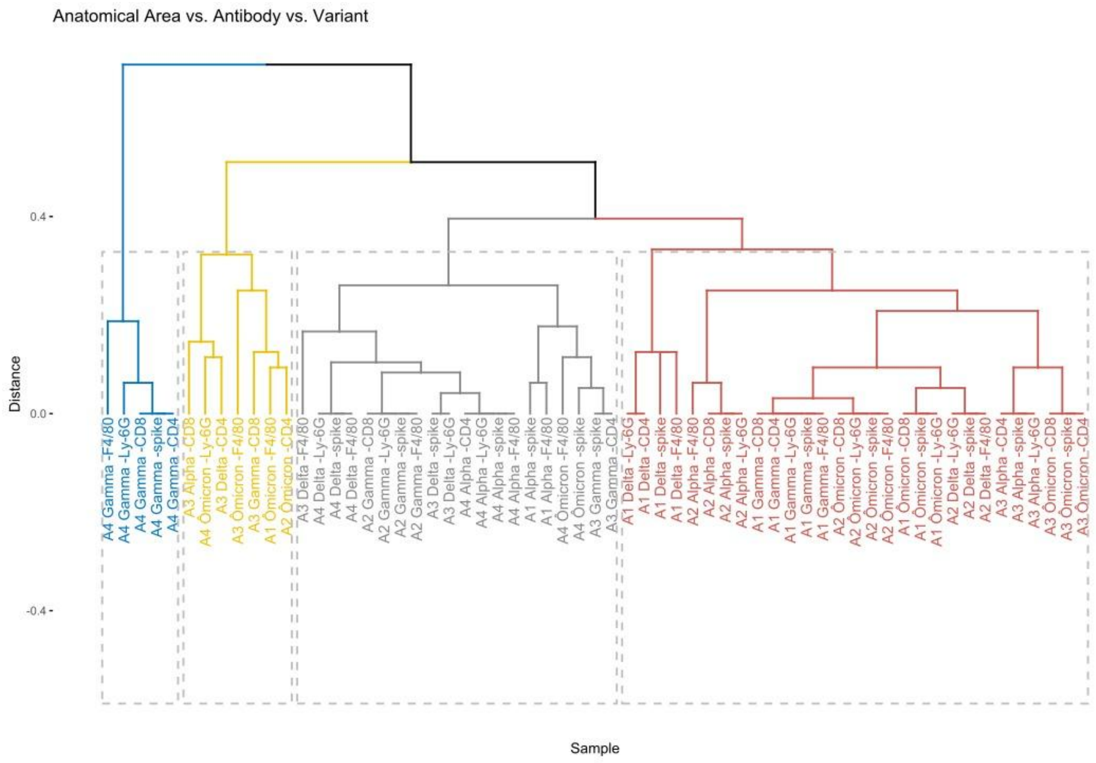
Anatomical Area vs. Antibody vs. Variant
Distance
0.4
0.0
-0.4
Sample
A4 Gamma -F4/80
A4 Gamma -Ly-6G
A4 Gamma -CD8
A4 Gamma -spike
A4 Gamma -CD4
A3 Alpha -CD8
A4 Ômicron -Ly-6G
A3 Delta -CD4
A3 Ômicron -F4/80
A3 Gamma -CD8
A1 Ômicron -F4/80
A2 Ômicron -CD4
A3 Delta -F4/80
A4 Delta -Ly-6G
A4 Delta -spike
A4 Delta -F4/80
A2 Gamma -CD8
A2 Gamma -Ly-6G
A2 Gamma -spike
A2 Gamma -F4/80
A3 Delta -spike
A3 Delta -Ly-6G
A4 Alpha -CD4
A4 Alpha -Ly-6G
A4 Alpha -spike
A4 Alpha -F4/80
A1 Alpha -spike
A1 Alpha -F4/80
A4 Ômicron -F4/80
A4 Ômicron -spike
A3 Gamma -spike
A3 Gamma -CD4
A1 Delta -Ly-6G
A1 Delta -CD4
A1 Delta -spike
A1 Delta -F4/80
A2 Alpha -F4/80
A2 Alpha -CD8
A2 Alpha -spike
A2 Alpha -Ly-6G
A1 Gamma -CD8
A1 Gamma -CD4
A1 Gamma -Ly-6G
A1 Gamma -spike
A1 Gamma -F4/80
A2 Ômicron -CD8
A2 Ômicron -Ly-6G
A2 Ômicron -spike
A2 Ômicron -F4/80
A1 Ômicron -CD8
A1 Ômicron -spike
A1 Ômicron -Ly-6G
A2 Delta -Ly-6G
A2 Delta -spike
A2 Delta -F4/80
A3 Alpha -CD4
A3 Alpha -spike
A3 Alpha -Ly-6G
A3 Ômicron -CD8
A3 Ômicron -spike
A3 Ômicron -CD4
