# Supplementary material for: SARS-CoV-2 Gamma Variant In Vivo Model Demonstrates Severe Pathogenicity and Unique Transcriptomic Signatures Compared to Alpha, Delta, and Omicron Variants
Source: Microorganisms. 2026 Oct 20;14(10):2400. doi: 10.3390/microorganisms14102400 (PMC13707728; doi:10.3390/microorganisms14102400)
Supplement: Supplementary file 1 [file microorganisms-14-02400-s001.zip › Supplementary_Figure S3.pdf]

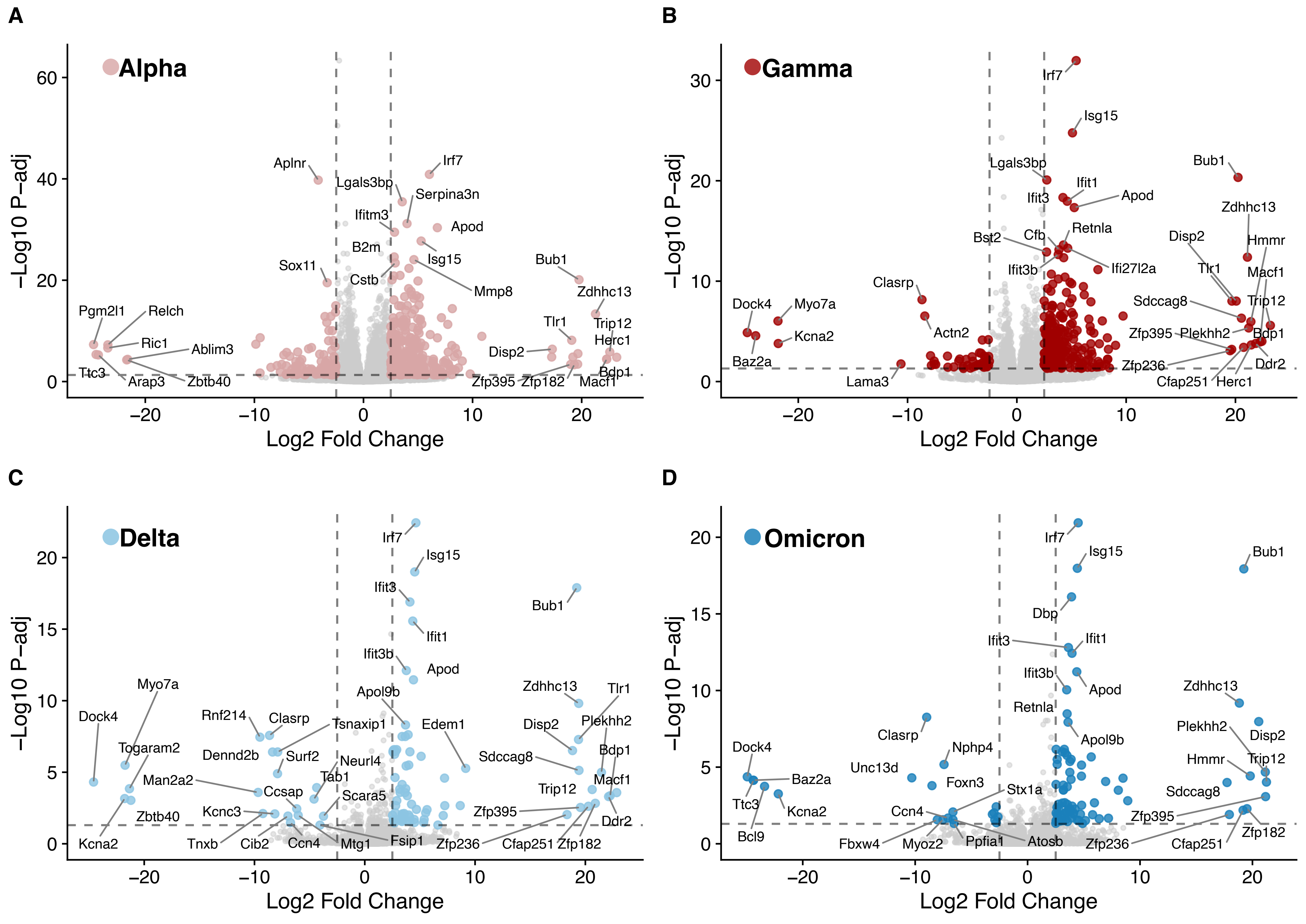
A
Alpha
−Log10 P−adj
Log2 Fold Change
Aplnr
Irf7
Lgals3bp
Serpina3n
Ifitm3
Apod
B2m
Isg15
Sox11
Cstb
Mmp8
Bub1
Zdhhc13
Pgm2l1
Relch
Ric1
Ablim3
Ttc3
Arap3
Zbtb40
Tlr1
Trip12
Herc1
Disp2
Zfp395
Zfp182
Macf1
Bdp1
B
Gamma
Irf7
Isg15
Lgals3bp
Ifit1
Ifit3
Apod
Bub1
Zdhhc13
Bst2
Cfb
Retnla
Ifit3b
Ifi27l2a
Disp2
Hmmr
Tlr1
Macf1
Clasrp
Dock4
Myo7a
Kcna2
Baz2a
Actn2
Lama3
Sdccag8
Trip12
Zfp395
Plekhh2
Bdp1
Zfp236
Ddr2
Cfap251
Herc1
C
Delta
Irf7
Isg15
Ifit3
Bub1
Ifit1
Ifit3b
Apod
Apol9b
Zdhhc13
Tlr1
Myo7a
Dock4
Rnf214
Clasrp
Tsnaxip1
Edem1
Disp2
Plekhh2
Togaram2
Dennd2b
Surf2
Neurl4
Sdccag8
Bdp1
Tab1
Macf1
Man2a2
Ccsap
Scara5
Trip12
Zbtb40
Kcnc3
Zfp395
Ddr2
Kcna2
Tnxb
Cib2
Ccn4
Mtg1
Fsip1
Zfp236
Cfap251
Zfp182
D
Omicron
Irf7
Isg15
Bub1
Dbp
Ifit3
Ifit1
Ifit3b
Apod
Zdhhc13
Retnla
Clasrp
Apol9b
Plekhh2
Disp2
Dock4
Nphp4
Unc13d
Hmmr
Trip12
Baz2a
Foxn3
Stx1a
Sdccag8
Ttc3
Kcna2
Ccn4
Zfp395
Bcl9
Fbxw4
Myoz2
Ppfia1
Atosb
Zfp236
Cfap251
Zfp182
